# Intraspecies competition among *Salmonella enterica* isolates in the lettuce leaf apoplast

**DOI:** 10.3389/fpls.2024.1302047

**Published:** 2024-01-30

**Authors:** Cristián Jacob, Joseph Student, David F. Bridges, Weiping Chu, Steffen Porwollik, Michael McClelland, Maeli Melotto

**Affiliations:** ^1^ Departamento de Ciencias Vegetales, Facultad de Agronomía y Sistemas Naturales, Pontificia Universidad Católica de Chile, Santiago, Chile; ^2^ Department of Plant Sciences, University of California, Davis, Davis, CA, United States; ^3^ Horticulture and Agronomy Graduate Program, University of California, Davis, Davis, CA, United States; ^4^ Plant Biology Graduate Group, University of California, Davis, Davis, CA, United States; ^5^ Department of Microbiology and Molecular Genetics, University of California, Irvine, Irvine, CA, United States

**Keywords:** fresh produce, food safety, plant defense, pathogen contamination, competition assay, apoplastic nutrient, leafy green, genetic barcoding

## Abstract

Multiple *Salmonella enterica* serovars and strains have been reported to be able to persist inside the foliar tissue of lettuce (*Lactuca sativa* L.), potentially resisting washing steps and reaching the consumer. Intraspecies variation of the bacterial pathogen and of the plant host can both significantly affect the outcome of foliar colonization. However, current understanding of the mechanisms underlying this phenomenon is still very limited. In this study, we evaluated the foliar fitness of 14 genetically barcoded *S*. *enterica* isolates from 10 different serovars, collected from plant and animal sources. The *S*. *enterica* isolates were vacuum-infiltrated individually or in pools into the leaves of three- to four-week-old lettuce plants. To estimate the survival capacity of individual isolates, we enumerated the bacterial populations at 0- and 10- days post-inoculation (DPI) and calculated their net growth. The competition of isolates in the lettuce apoplast was assessed through the determination of the relative abundance change of barcode counts of each isolate within pools during the 10 DPI experimental period. Isolates exhibiting varying apoplast fitness phenotypes were used to evaluate their capacity to grow in metabolites extracted from the lettuce apoplast and to elicit the reactive oxygen species burst immune response. Our study revealed that strains of *S*. *enterica* can substantially differ in their ability to survive and compete in a co-inhabited lettuce leaf apoplast. The differential foliar fitness observed among these *S*. *enterica* isolates might be explained, in part, by their ability to utilize nutrients available in the apoplast and to evade plant immune responses in this niche.

## 1 Introduction

Foodborne diseases are caused by a variety of biological hazards including viruses, bacteria, protozoa, and helminths. Particularly, diarrheal disease-causing viruses (Norovirus) and bacteria (*Campylobacter* spp., enteropathogenic *Escherichia coli*, and non-typhoidal *Salmonella enterica*) are responsible for most of the foodborne illnesses and deaths worldwide (World Health Organization, 2015). Comparative genomic studies of pathogenic *E*. *coli* and *S*. *enterica* isolates have revealed substantial intraspecific variation, such as structural and functional features, that might influence the differences in their epidemiology (de Moraes et al., 2018; Nguyen et al., 2018; Sharma et al., 2019). *Salmonella enterica* subsp. *enterica* comprises more than 2,500 serovars that often affect the human population in a seasonal-, geographic-, and demographic-dependent manner (CDC, 2013). Freshly eaten fruits and vegetables, particularly leafy greens, are frequently vectors of serovars of *S. enterica* (Lynch et al., 2009; Callejón et al., 2015; Bennett et al., 2018; Turner et al., 2019; Marshall et al., 2020). Between 1998 and 2013, over 10,000 people in the USA were confirmed to have *S. enterica* infections after consuming contaminated produce. A total of 34 serovars were identified as the causative agents with Newport, Enteritidis, Typhimurium, Javiana, and Saintpaul ranking as the top five (Bennett et al., 2018).

Recent studies have revealed the extensive capacity of human pathogenic bacteria to attach, internalize, and/or persist in various tissues of different plant species (Han and Micallef, 2014; Cui et al., 2018; Erickson and Liao, 2019; Roy and Melotto, 2019; Wong et al., 2019; Merget et al., 2020). However, successful colonization by *S. enterica* and other pathogens varies depending on parameters such as the plant species, the location of the tissue, and the serovar or strain of bacteria. For example, Cui et al. (2018) inoculated alfalfa, tomato, fenugreek, and tomato seeds with four *S. enterica* serovars (Baildon, Cubana, Montevideo, and Stanley) and found that populations of Cubana and Baildon were significantly higher than those of the other serovars in tomato and fenugreek seedlings, respectively. In contrast, there was no significant difference between populations of the four serovars in alfalfa or lettuce seedlings. In addition, the serovar populations varied significantly between the root, seed coat, stem, and cotyledons of sprouts three days after germination. Furthermore, the survival of 43 *S. enterica* strains from 29 serovars exhibited significant variation after surface inoculation of 2- to 3-week-old lettuce and tomato seedlings in microcentrifuge tubes containing a nutritional medium (Wong et al., 2019).

*Salmonella* is a member of the *Enterobacteriaceae* family, which possesses a conserved genomic backbone (Alnajar and Gupta, 2017). *Enterobacteriaceae* includes human and plant pathogens, both of which can attach to and internalize into plant tissues (Brandl and Mandrell, 2002; Kroupitski et al., 2011; Roy et al., 2013). However, enterobacterial human pathogens like *S. enterica* are not known to contain plant-related virulence factors, such as phytotoxins and cell wall-degrading enzymes, capable of causing symptoms or disease on plants (Toth et al., 2006). Nevertheless, serovars of *S. enterica* can colonize plants and elicit an immune response (Garcia et al., 2014; Jechalke et al., 2019; Jacob and Melotto, 2020). Therefore, serovars of *S. enterica* might be utilizing a set of bacterial functions distinct from those encoded by their virulence regulons known to be required for human colonization and from those used by phytopathogens (Teplitski and de Moraes, 2018).

Our understanding of the mechanism underlying the intraspecies variation in the ability of human pathogenic bacteria to survive in various plant environments is still limited. In this study, we evaluated the capacity of 14 *S*. *enterica* isolates from 10 different serovars, collected from plant and animal sources, to persist and compete for survival in the leaf apoplast of two lettuce cultivars that have contrasting responses to bacterial colonization. The lettuce leaf apoplast was utilized as *S. enterica* can internalize into and reside in this habitat (Kroupitski et al., 2011; Roy and Melotto, 2019). Bacterial fitness competition assays were performed using clones distinguished by barcodes in their genomes (Santiviago et al., 2009; Silva-Valenzuela et al., 2016; Porwollik et al., 2018). These assays revealed that strains of *S. enterica* can substantially differ in their ability to compete in a co-inhabited lettuce leaf apoplast. The differential foliar fitness observed among these *S. enterica* isolates might be explained, in part, by their ability to utilize nutrients available in the apoplast and to evade plant immune responses in this niche.

## 2 Materials and methods

### 2.1 Plant material and growth conditions

Seeds of the lettuce cultivars (*Lactuca sativa* L.) Red Tide (RT) and Lollo Rossa (LR) were germinated in pre-soaked paper for 2 days at 20°C, then transferred to peat moss pellets (42 mm, Jiffy^®^ 7, Canada) and grown at 18 ± 2°C and 80 ± 10% relative humidity, with a 12-hour photoperiod and light intensity of 240 ± 10 µmol m^-2^ sec^-1^. At 10 days after germination, 0.05 g/plant of fertilizer (Multi-Purpose 19-11-21, Peters^®^Excel, OH, USA) was dissolved in the irrigation water. Three- to four-week-old plants were used for inoculations and four plants (each representing one biological replicate) were used for each sampling point (n=4). After inoculation, plants were transplanted into pots containing growing substrate (Redi-earth plug and seedling mix, Sun Gro, MA, USA) and maintained in the same growing conditions.

### 2.2 Bacterial isolates and barcoding

To assess potential competition among outbreak-associated *S*. *enterica* strains in the leaf environment, we attempted to introduce a DNA barcode into a neutral location in 20 *S*. *enterica* isolates collected from different animal and plant sources, of which 14 had successful recombination events and hence stable insertions. Therefore, barcodes were successfully introduced into 14 isolates representing 10 *S*. *enterica* serovars (**Table 1**). The abbreviations used for the isolates are composed of the letter S (for *Salmonella*), followed by the first letters of the serovar name and a number to indicate different isolates of the same serovar. Construction of clones with 21-base neutral barcodes flanked by the Illumina sequencing primer sequences was conducted using primers and strategies previously described in Porwollik et al. (2018). In brief, we PCR-amplified a kanamycin resistant cassette from pCLF4 with primers that also added an N_21_ string flanked by the sequences of the Illumina sequencing primers. To this PCR product we added, by PCR, 120-base sequences at each end that are homologous to the beginning and end of the bacterial chromosomal *phoN* gene, which is considered a neutral location for an insertion because of a lack of a mutant phenotype (Weening et al., 2005). The 120-base homology ensured that the required 30 bases of identity to allow Lambda-red recombination were present in almost all strains regardless of sequence divergence. The primer sequences, including 20 bases of homology with the pCLF4 KanR PCR product at the 3’ of each primer, were phoN-1: 5’-CTACCACTGATCGTAGCTAAATATACATCAGCAGAAACAGTGCAACCCTTTCATTCTCCTGAAGAATCAGTGAACAGTCAGTTCTACTTACCACCACCGCCAGGTAATGATGATCCGGCTTGTGCAGGCTGGAGCTGCTTC-3’ and phoN-2: 5’-ACGCAGTTGCACTTCCTTTCATTTGCTGTGGCCAGTTTGCGGGAAGACTTTCACCTTCAGTAATTAAGTTTGGGGTGATCTTCTTTACTCAATAAATTATTTTTGTCGTTCAGCTCCTCACGGACTTTTGCCAGTGACTTCTGAACATATGAATATCCTCCTTAG-3’. In serovars Anatum and Montevideo, sequence divergence precluded success with these primers and primers homologous to their specific *phoN* sequence were used, instead. These primers were shorter because the specific *phoN* sequence used in the primer design obviated the need for tolerance of sequence diversity. For the Anatum isolate, both primers were replaced, phoN-1SA: 5’-TGAGTAAAGAAGATCACCCCGAACTTAATTACTGAAGGTGCAGGCTGGAGCTGCTTC-3’ and phoN-2SA: 5’-GGAAGACTTTCACCTTCAGTAATTAAGTTCGGGGTGATCTTCTTTCATATGAATATCCTCCTTAG-3’. For the Montevideo isolate, only one primer was replaced, phoN-1SM: 5’-CCGGAGTGAGTCTTTATGAAAAGTCGTTATTTACTATTTTTTCTAGTGCAGGCTGGAGCTGCTTC-3’, and used with primer phoN-2.

**Table 1:** Description of *Salmonella enterica* isolates used for fitness competition assays.

Serovar	Abbreviation	Isolate designation	Source	Barcoded clones (#)	Genome assembly accession
Anatum	SA	11TTU577B	Cattle	8	-
Enteritidis	SE	LJH0704	**Sprout water**	8	-
Hartford	SH	LJH590 (H0778)	**Orange juice**	5	-
Kottbus	SK	LJH0706 (01A-2858)	**Alfalfa sprout**	8	-
Michigan	SMi	LJH0553	**Tomato**	8	-
Montevideo	SM-1	SAL2345	**Lettuce head**	5	ASM24078v2
Montevideo	SM-2	LJH0519 (G4639)	**Tomato**	8	ASM23853v2
Montevideo	SM-3	11TTU1694B	Cattle	8	-
Oranienburg	SO	LJH0705 (97A-2285)	**Alfalfa sprout**	6	-
Poona*	SP-1	00A-3279	Human	7	-
Poona	SP-2	01A-242	Human	8	-
Rubislaw	SR	LJH588 (S2833)	**Orange juice**	7	-
Typhimurium	STm-1	SPN463	Cattle	8	-
Typhimurium	STm-2	14028s	Chicken	92	ASM2216v1

Isolates from plant sources were associated with disease outbreaks linked to that commodity. Isolates with an LJH number are from the Western Center for Food Safety collection at UC Davis and strains with a TTU number are from the Texas Tech University strain collection. Plant-based sources are shown in bold letters.

*Strain obtained from the US Department of Health, which was isolated from patient involved in an outbreak involving cantaloupe.

During introduction of the barcode into the strains, recombination involves a single molecule of the PCR product containing the KanR cassette. Therefore, one unique sequence of the N_21_ string (the barcode) was involved in the generation of each clone. The unique barcodes in each strain are flanked by 30-base sequences that are homologous to the sequences of the two standard Illumina sequencing primers, allowing barcode identification in standard Illumina sequencing reactions. The barcodes were amplified with primers containing indexes to uniquely identify each sample and the two Illumina sequencing primer sequences. The PCR products from all samples were purified together and the barcodes and indexes identifying each strain and sample, respectively, were sequenced using an Illumina sequencer following a standard manufacturer’s procedure.

*Salmonella enterica* barcoded clones (**Table 1**) were either inoculated as mixes of clones of a single isolate or combined into pools of up to five different isolates (**Table 2**). Inoculations with pools allowed the measurement of the relative abundance of each isolate when competing in the lettuce leaf apoplast.

**Table 2:** Description of the pools created with *Salmonella enterica* isolates.

Category	Pool #	Strains in the pool
SM-1 with isolates from animals	P01	SM-1 and SA
	P02	SM-1 and SP-2
	P03	SM-1 and SM-3
	P04	SM-1 and STm-2
	P05	SM-1 and SP-1
SM-1 with isolates from plants	P06	SM-1 and SM-2
	P07	SM-1 and SO
Animal pool	P08	SA, SM-3, SP-2, and STm-2
SM-1 with isolates from plants and animals	P09	SM-1, SP-1, SP-2, SR, and STm-2
Isolates from sprouts (SE, SO, and SK) in complex pools	P10	SE, SH, SK, SMi, and STm-1
	P11	SE, SH, SM-2, SO, and STm-1

### 2.3 Bacterial inoculum preparation

*Salmonella enterica* isolates (**Table 1**) were grown in low-salt Luria Bertani (LSLB) medium at 28°C. To maintain the proportion of barcoded clones in each isolate, bacterial stocks were thawed on ice, mixed thoroughly, and 10 μL were placed into a culture tube containing 5 mL of LSLB medium for overnight growth (**Supplementary Figure S1**). LSLB medium was supplemented with either 15 μg/mL tetracycline or 60 μg/mL kanamycin, as appropriate. Upon reaching an OD_600_ of 0.7 to 0.9, bacterial cells were collected by centrifugation and a two-step dilution process was used to prepare the inoculum as described by Oblessuc and Melotto (2020) for a final concentration of 1 x 10^3^ CFU/mL. To make inocula with pools of multiple *S. enterica* isolates (**Table 2**), equal amounts of each isolate were added. Silwet^®^ L-77 (PhytoTech Labs, Lenexa, KS) was added to the inoculum to a final concentration of 0.01%. To confirm adequate bacterial concentration in the inoculum, the bacterial population was enumerated using serial-dilution plating.

### 2.4 Leaf inoculation and bacterial enumeration

Leaves were vacuum-infiltrated with inoculum and the apoplastic bacterial population size (CFU/g foliar tissue) was estimated through enumeration using serial-dilution plating as previously described (Oblessuc and Melotto, 2020; **Supplementary Figure S1**). The bacterial population size was estimated at 0 and 10 days post-inoculation (DPI). Sampling at 0 DPI occurred once water soaking disappeared from the leaves at roughly 3 hours post inoculation. This sampling point was used to ensure that the bacterial population size across leaf samples was uniform at the time of inoculation. At 10 DPI leaves samples were surface sterilized with 70% ethanol for 1 min and then rinsed in sterile deionized water (SDW) for 1 min. For all time points, the second true leaf was sampled and the fresh weight was taken on an analytical balance. The leaf was macerated, and the bacteria were recovered in a phosphate-buffered saline solution (8 g/L NaCl, 0.2 g/L KCl, 1.44 g/L Na_2_HPO_4_, 0.24 g/L KH_2_PO_4_). The resulting suspension was used for plating or the expansion step for the 10 DPI sampling.

Bacterial population net growth was calculated as the Log_2_ ratio between the population at 0 and 10 DPI. Changes in population size over time were defined as positive, neutral, or negative net growth according to the definition of bacterial population growth as “the number of viable cells versus time” including the stationary and death phases (Madigan et al., 2014).

### 2.5 Bacterial recovery from leaves

Bacterial samples were prepared for sequencing both from the inoculum medium and from the leaves at 10 DPI (**Supplementary Figure S1**). Before vacuum infiltration, 480 μL of the inoculum suspensions, at a concentration of 1 x 10^8^ CFU/mL, were transferred to cryogenic vials, mixed with 320 μL of sterile 50% glycerol, frozen in liquid nitrogen, and stored at -80°C. Due to the relatively low bacterial concentrations recovered from the leaf tissues at 10 DPI, an expansion step was required to attain adequate bacterial levels to capture enough sequencing reads for downstream analyses. To this end, 100 µL of the leaf macerate used for bacterial enumeration was spread on LSLB agar medium. After overnight incubation at 30°C, 1.3 mL of LSLB broth was added to the solid LSLB culture plate and bacteria were recovered with a spreader. Following recovery, 480 µL of this suspension was placed in a cryogenic vial, frozen, and stored at -80°C.

### 2.6 Bacterial barcode sequencing

Sample processing and transposon sequencing was performed as previously described (de Moraes et al., 2017; Jayeola et al., 2020). In brief, an aliquot of the bacteria recovered from leaves (about 2 x 10^7^ cells) was spun down and washed three times with water and then the pellet was resuspended in 20 µL of lysis buffer (5 mM Tris [pH 8.0], 0.5 mM EDTA, 0.05% Triton X-100), supplemented with 100 ug proteinase K and incubated at 55°C for one hour followed by ten minutes at 95°C. An aliquot of 5 µL was subjected to PCR with standard Illumina primers, with unique indexes for each sample using Illumina protocols. The barcodes were sequenced on 10% of a single NovaSeq6000 lane and enumerated using custom Perl scripts.

### 2.7 Bacterial fitness competition assay in the leaf apoplast

Following a similar approach used by Porwollik et al. (2018) to study population dynamics during *Salmonella* colonization of cattle, we determined the relative fitness of each isolate inside the leaf by comparing the changes in the relative abundance of the population of each isolate within a pool (**Supplementary Figure S1**). Read counts obtained from the sum of barcoded clones of each *S*. *enterica* isolate in a pool (**Supplementary Dataset S01**) were used to calculate the population change over time using the following formula: Log_2_ (population relative abundance at 10 DPI/population relative abundance in the inoculum). Four plants were used for each sample point for all experiments (n=4) and the inoculum was sequenced from one sample. Statistically significant changes in the mean relative abundance of isolates indicate the existence of competition among the isolates when they co-exist in the lettuce leaf.

### 2.8 Apoplastic wash fluid extraction

Water-soluble metabolites within the apoplast of RT lettuce were recovered through the extraction of apoplastic wash fluid (AWF), as described by (O’Leary et al., 2014). The two youngest fully expanded leaves of 3-week-old RT plants were cut, rinsed with SDW for 1 min to remove leaf surface contaminants, and placed into a 60 mL syringe to infiltrate SDW containing M9 salts (Na_2_HPO_4_ 12.8 g l^-1^, KH_2_PO_4_ 3 g l^-1^, NaCl 0.5 g l^-1^, and NH_4_Cl 1 g l^-1^). Surface moisture of fully soaked leaves was gently removed with paper towel and leaves were rolled around 1 mL pipet tips, wrapped with parafilm, and placed into 50 mL centrifuge tubes. Tubes were centrifuged at 267 x *g* for 8 min at 4°C to collect AWF that was immediately filter-sterilized using a 0.22 µm syringe filter (Restek, PA, USA) and stored at -80°C.

### 2.9 Bacterial growth in AWF

Utilization of AWF as growth medium was performed as described by Montano et al. (2020). Briefly, 5 µL of an 0.02 OD_600_ inoculum from each *S. enterica* isolate was added to 195 µL of each medium (M9 salts minimal medium, LSLB rich medium, and AWF from RT) using a 96-well microtiter plate. Growth curves were obtained through OD_600_ measurements of cultures incubated on a plate reader (Synergy H1 Hybrid Multi-Mode Reader, Biotek, Winooski, VT, USA), until the stationary phase of bacterial growth was reached. The average maximum growth rate (µmax) was estimated using the GrowthRates package in R (R Core Team, 2020). The experiment included three biological replicates from separately grown batches of plants (n=3).

### 2.10 ROS burst assay

Apoplastic reactive oxygen species (ROS) burst was quantified as previously described (Smith and Heese, 2014). Unlike RT, LR plants produce a strong ROS burst in response to the *S. enterica* isolate STm 14028s (Jacob and Melotto, 2020); thus, they were used for this assay. Briefly, leaf discs (5 mm in diameter) from the third leaf of 3.5-week-old plants were placed, abaxial side down, into individual wells of a white 96-well plate (Nunc-Immuno™ MicroWell™ 96-well polystyrene plates; Sigma-Aldrich, Darmstadt, Germany) containing 200 µL of SDW and incubated for 20-24 hours at constant light and 22°C to reduce the wounding response. SDW was replaced with 150 µL of elicitation solution, containing 20 µg of horseradish peroxidase (MilliporeSigma, Burlington, MA, USA) and 32 µg of luminol (Millipore Sigma, Burlington, MA, USA) per mL of SDW, with or without 5 x 10^8^ CFU/mL of heat-killed bacterial mixes. Heat-killed bacteria (incubated at 100°C for 10 min) were used to avoid ROS production based on any virulence factors produced by live bacteria. The 96-well plate was immediately placed in a microplate reader (Synergy H1 Hybrid Multi-Mode Reader, Biotek, Winooski, VT, USA) to measure luminescence every 2 minutes for 90 minutes. Each treatment consisted of 16 leaf discs collected from the third leaf of three separately grown plants (n=3).

### 2.11 Statistical analysis

Data analyses were conducted in R (R Core Team, 2020). To assess the effect of an *S. enterica* isolate on dependent variables such as changes in the relative abundance, ROS burst peak height, µmax, and highest OD, data were subjected to analysis of the variance (ANOVA). To this end, the lm function was used to create the linear models that were entered into the aov function to run ANOVA. Subsequently, multiple comparison of means was conducted through Tukey’s test with the HSD.test function (agricolae package), considering α=0.05. For pair-wise comparisons (*i*.*e*., bacterial populations at 0 DPI vs. 10 DPI and changes in the relative abundance of SM-1 vs. other isolates), Student´s *t*-tests were used (t.test function, ggpubr package). Box plots were built with the ggplot2 package. Each box shows the interquartile range (distance between the first and the third quartiles), the line in the box represents the median, whiskers represent the smallest and highest data points, and the dots outside the box correspond to outliers.

## 3 Results

### 3.1 *Salmonella enterica* net growth depends on the lettuce cultivar and bacterial isolate

**Figure 1:**
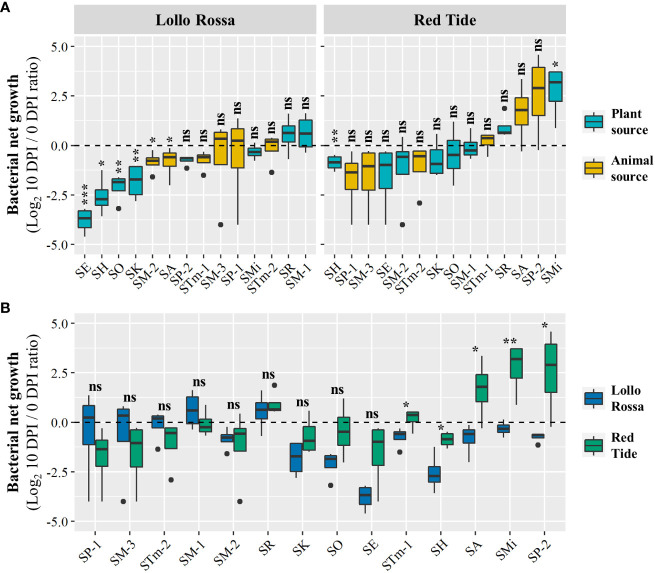
Net growth of the *Salmonella enterica* isolates in the leaf apoplast of the lettuce cultivars Lollo Rossa and Red Tide over the period of 10 days. Graphs show the effect of bacterial **(A)** and plant **(B)** genotypic variation on the bacterial net growth of individually inoculated isolates. Results are shown as mean ratio between the bacterial population at 10 days post-inoculation (DPI) compared to that of in the day of inoculation (0 DPI). Plot center lines show the medians; box limits indicate the 25^th^ and 75^th^ percentiles, and whiskers extend to minimum and maximum data points. Significant differences between the bacterial population at 0 and 10 DPI **(A)** and between the bacterial net growth in Lollo Rossa and Red Tide **(B)** were assessed by Student’s *t*-test (ns, not significant, *p<0.05, **p<0.01, ***p<0.001). Four plants were used for each data point (n=4).

We have recently reported that the human pathogenic bacteria *E*. *coli* O157:H7 and *S. enterica* serovar Typhimurium 14028s can survive significantly better in the lettuce cultivar Red Tide (RT) when compared to the cultivar Lollo Rossa (LR), and these phenotypes were associated with variations in the plant defense responses (Jacob and Melotto, 2020). Thus, we used these contrasting lettuce genotypes to assess the ability of different *S. enterica* isolates (**Table 1**) to persist in their leaf apoplast. Overall, the LR apoplast was a less suitable environment for the isolates than the apoplast of RT. The range of bacterial net growth in LR was from an average of Log_2_ -3.8 [ ± 0.3 standard error (SE)] to Log_2_ 0.5 ( ± 0.4 SE), whereas in RT it ranged from Log_2_ -1.6 ( ± 0.8 SE) to Log_2_ 2.7 (± 0.6 SE) (**Figure 1A**). In the LR plants, all tested isolates had either statistically significant reductions (*i*.*e*., negative net growth) or non-significant changes in population (*i*.*e*., neutral net growth). In comparison, 12 out of the 14 tested isolates had neutral net growth in RT, with SH and SMi having net negative and net positive growths, respectively (**Figure 1A**). Furthermore, we observed that the isolates showing the strongest decrease in their populations during the 10 DPI period in LR were from plant sources (SE, SO, SK, and SH), while in RT the isolates from animal and plant sources were spread seemingly randomly along the range of variation in net growth (**Figure 1A**). As bacterial enumeration data were taken from both lettuce genotypes at the same time, we were able to statistically compare the cultivars for bacterial net growth phenotype (**Figure 1B**). Five out of the 14 isolates (STm-1, SH, SA, SMi, and SP-2) exhibited significant differences in their bacterial net growth in RT and LR (**Figure 1B**). However, only SA and SMi showed different bacterial net growth outcomes. For instance, SMi exhibited a neutral and significant positive net growth in LR and RT, respectively (**Figure 1A**). In addition, through CFU enumeration we quantified the bacterial populations of each pool of *S. enterica* isolates at 0 and 10 DPI (**Supplementary Figure S1**), estimating the total population net growth of each pool in the lettuce cultivars (**Figure S2**). In general, the relative survival capacity of the isolates was better in RT as compared to LR (**Supplementary Figure S2**). In LR, six pools exhibited neutral net growth and five pools exhibited significantly decreased net growth during the experimentation period (**Supplementary Figure S2A**). By contrast, in RT, five pools exhibited significant positive net growth and five pools showed no significant changes in their populations over time (**Supplementary Figure S2A**). Six out of the 11 pools of *S. enterica* isolates exhibited significant differences in their total population net growth in RT and LR (**Supplementary Figure S2B**). Differences in the population kinetics of each pool in the lettuce genotypes varied from significantly negative net growth in LR and neutral in RT (P04), neutral in LR and significantly positive in RT (P05 and P09), to negative in LR and positive in RT (P08 and P10). Altogether, these findings indicate intraspecies variation among *S*. *enterica* isolates in their capacity to persist in the foliar apoplast of two lettuce cultivars and support the notion that RT can be more suitable for *S. enterica* survival than LR.

### 3.2 Competition capacity of SM-1 varies according to the co-inoculated *S*. *enterica* isolate

**Figure 2:**
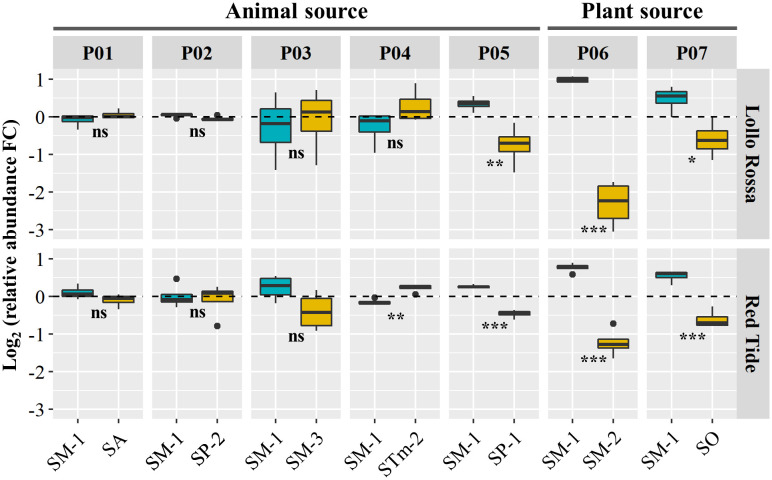
Paired competition between SM-1 (*Salmonella enterica* ser. Montevideo, isolated from head lettuce) and *S*. *enterica* strains collected from animal (Pools 01 – 05) or plant (Pools 06 and 07) sources. The inoculum containing each pool was vacuum infiltrated into the leaf apoplast of the lettuce cultivars Lollo Rossa and Red Tide. Competition was assessed based on the Log_2_ fold change (FC) between the isolates’ relative abundance in the inoculum and in leaves at 10 days post inoculation. Plot center lines show the medians; box limits indicate the 25^th^ and 75^th^ percentiles, and whiskers extend to minimum and maximum data points. Statistically significant differences between the mean (n=4) changes in relative abundance of the *S*. *enterica* isolates in each pool were assessed by Student’s *t*-test (ns, not significant, *p<0.05, **p<0.01, ***p<0.001).

The colonization of the foliar tissue by human pathogenic bacteria might be substantially affected by the interaction with other microbes residing in this niche (Brandl et al., 2013). Thus, after establishing the capacity of individual *S. enterica* isolates to survive in the lettuce leaf apoplast, we assessed the performance of the strains in a co-inhabited environment through paired competition assays. The isolate SM-1 (ser. Montevideo), which was collected from head lettuce, was selected to compete with isolates from either an animal or a plant source (Pools 01 – 07; **Table 2**). Read counts of the barcoded strains (**Supplementary Dataset S01**) were used to estimate the relative abundance of each *S*. *enterica* isolate within each pool in the inoculum and *in planta* in the leaf apoplast at 10 DPI (**Supplementary Figure S3**). Then, we calculated the Log_2_ value of the ratio between the relative abundance at 10 DPI versus at the inoculum to estimate the competition capacity of the isolates (**Figure 2**).

The change in relative abundance of SM-1 was not statistically different from three of the five strains isolated from animal sources (SP-2, SA, and SM-3) in both lettuce genotypes (**Figure 2**). In contrast, the relative abundance of STm-2 strain was significantly higher than that of SM-1 in RT and SM-1 significantly outcompeted SP-1 in both RT and LR (**Figure 2**). While both SP-1 and SP-2 are isolates from humans belonging to the Poona serovar, SM-1 was only able to outcompete SP-1 (**Figure 2**). As the SA, SP-2, SM-3, and STm-2 strains were all from animal sources and exhibited overall similar levels of fitness when co-residing with SM-1 (**Figure 2**), we next competed them all together in Pool 08 for 10 days in the leaf apoplast (**Supplementary Figure S4**). We confirmed that the relative abundance of these isolates did not vary significantly in LR, whereas SP-2 and SA outcompeted SM-3 and STm-2 in RT (**Supplementary Figure S4**), indicating that the lettuce cultivar influences the intraspecies competition of *S. enterica* in lettuce leaves.

When competing with isolates from plant sources, the SM-1 isolate showed significant advantages, regardless of the lettuce genotype (**Figure 2**). SM-1 relative abundance was significantly higher than that of SM-2 (from tomato) and SO (from alfalfa sprout) after 10 days of inoculation (**Figure 2**). For instance, in Pool 06 competition assays, SM-1 showed a positive change in relative abundance of Log_2_ 0.8 ( ± 0.06 SE) in RT and Log_2_ 1.1 ( ± 0.03 SE) in LR, while SM-2 exhibited negative values; Log_2_ -1.3 ( ± 0.2 SE) in RT and Log_2_ -2.3 ( ± 0.3 SE) in LR (**Figure 2**). Overall, these results revealed that the fitness of SM-1 varies in the paired competition assays according to the competing *S. enterica* isolate, generally outperforming isolates collected from plant sources but not those isolated from animal sources.

### 3.3 *Salmonella enterica* isolates competing in complex pools

**Figure 3:**
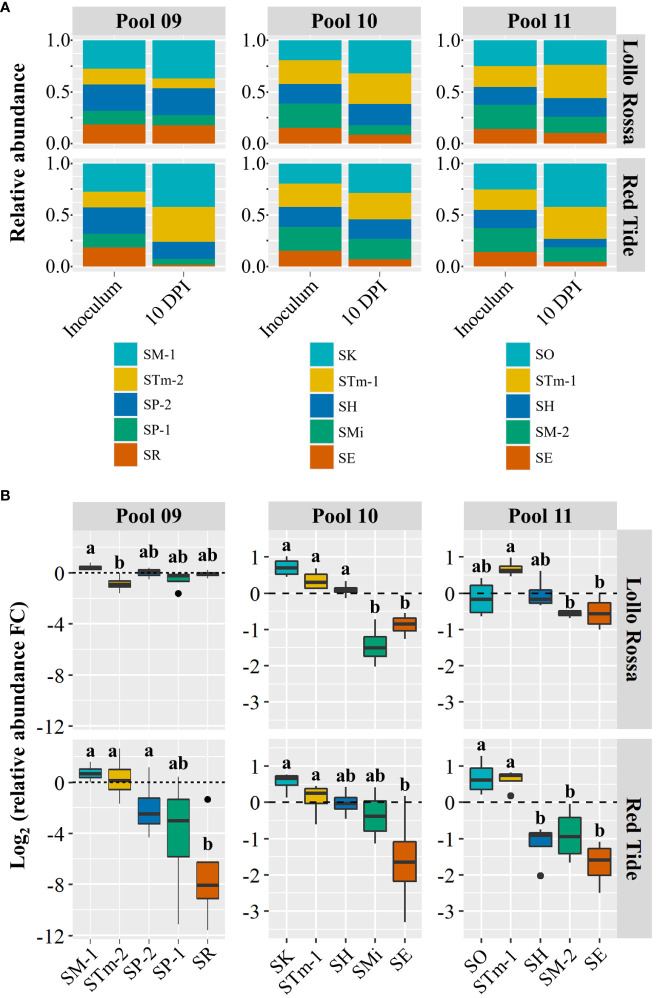
Intraspecies competition between *Salmonella enterica* isolates in complex pools. Relative abundance **(A)** and competition assessment **(B)** of the *Salmonella enterica* strains within Pool 09, Pool 10, and Pool 11 (**Table 2**). Pool 09 included SM-1 (*Salmonella enterica* ser. Montevideo, isolated from head lettuce) and strains collected from animal (SP-1, SP-2, and STm-2) or plant (SM-1 and SR) sources. Pool 10 and Pool 11 each combined five *S. enterica* isolates collected from alfalfa sprout (SO or SK), cattle (STm-1), orange juice (SH), tomato (SMi or SM-2), and sprout water (SE). The inoculum containing each combination of five *S*. *enterica* isolates was vacuum infiltrated into the leaf apoplast of the lettuce cultivars Lollo Rossa and Red Tide. Competition was assessed based on the Log_2_ fold change (FC) among the isolate relative abundance in the inoculum and in leaves at 10 days post inoculation. Plot center lines show the medians; box limits indicate the 25th and 75th percentiles, and whiskers extend to minimum and maximum data points. Different letters on top of adjacent boxes indicate significant statistical differences among the mean (n=4) changes in relative abundance of the *S*. *enterica* isolates as calculated with ANOVA followed by Tukey’s test (α=0.05).

To further evaluate the competition ability of *Salmonella* isolates, we conducted additional competition assays with complex pools that included combinations of five strains (Pools 09, 10, and 11; **Table 2**). First, we evaluated the performance of SM-1 in Pool 09 that contained strains recovered from animal (SP-1, SP-2, and STm-2) and plant (SR) sources. In LR, SM-1 was only able to significantly outcompete STm-2 (**Figure 3**), which contrasts with the pair-wise competition between the strains (**Figure 2**). The results from RT demonstrated that SM-1 could only outcompete SR, a strain isolated from orange juice, which was not used in the pair-wise comparisons. Additionally, SP-1, which was significantly outcompeted by SM-1 in the pair-wise comparisons (**Figure 2**), was found in similar relative abundances in this pool and SP-2 remained at a relative abundance comparable to SM-1 in both the pool and pair-wise assays (**Figures 2**, **3**). These results imply that SM-1 can perform equally or outcompete specific strains in the lettuce apoplast in both pair-wise interactions and in a complex pool containing five *S*. *enterica* isolates.

Next, we carried out competition assays using two complex pools that included barcoded *S. enterica* strains associated with outbreaks linked to alfalfa sprout production (SE, SK, and SO). To this end, we inoculated leaves of RT and LR with Pool 10 or Pool 11; each containing a combination of five isolates collected from alfalfa sprout (SO or SK), cattle (STm-1), orange juice (SH), tomato (SMi or SM-2), and sprout water (SE). In Lollo Rossa, SK, STm-1, and SH significantly outcompeted SMi and SE in pool 10, while in pool 11 only STm-1 was found at significantly higher levels than SM-2 and SE (**Figure 3**). In Red Tide, SK and STm-1 outcompeted only SE in pool 10, while in Pool 11, the sprout isolate SO and STm-1 were found at significantly higher levels than SH, SM-2, and SE (**Figure 3**). Interestingly, the two alfalfa sprout isolates, SO and SK, were consistently found in relative abundances comparable to STm-1 (a Typhimurium strain from cattle) despite SO belonging to the Oranienburg serovar and SK being a Kottbus. The only isolate consistently found at lower relative numbers than these serovars was SE that was obtained from sprout production water (**Figure 3**). Overall, these results demonstrate intraspecific variation in the competition fitness among strains of *S. enterica*, which is also dependent on the lettuce cultivar.

### 3.4 Growth in AWF and induction of plant immune response varies with *S*. *enterica* isolate

**Figure 4:**
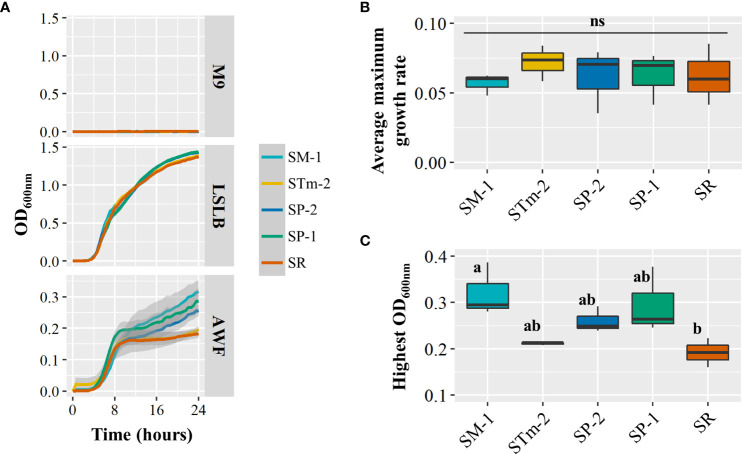
Growth performance of *Salmonella enterica* isolates collected from animal (SP-1, SP-2, and STm-2) and plant (SM-1 and SR) sources in the apoplastic wash fluid (AWF) recovered from the lettuce genotype Red Tide. **(A)** *Salmonella enterica* population kinetics during incubation in the negative (M9 salts minimal medium) and positive (Low Salt Luria Bertani medium) control media and in AWF. Shaded areas represent the standard error. Average maximum growth rate **(B)** and highest OD_600nm_ **(C)** of the *S. enterica* isolate mixes incubated in Red Tide AWF were estimated by using the GrowthRates package in the R software. The experiment included three biological replicates from batches of separately grown plants (n=3). Plot center lines show the medians; box limits indicate the 25^th^ and 75^th^ percentiles, and whiskers extend to minimum and maximum data points. Different letters on top of adjacent boxes indicate significant statistical differences as calculated with ANOVA followed by Tukey’s test (α=0.05). ns, not significant.

To gain some understanding of the mechanisms underlying the differential ability of *S. enterica* isolates to compete in the lettuce leaf apoplast, we used isolates in Pool 09 to assess two bacterial fitness traits, growth in AWF and the induction of plant immune responses (ROS burst). First, we extracted water-soluble nutrients from the apoplast of RT to use it as a medium because this cultivar offered an overall more suitable environment for bacterial survival (**Supplementary Figure S2**) and competition (**Figure 3**) of the isolates of the complex Pool 09 (**Table 2**). When incubated in the AWF medium, the different strains showed notable differences in growth patterns after the logarithmic phase (**Figure 4A**). No significant difference was detected in the maximum growth rate (µmax) among the isolates (**Figure 4B**). Interestingly, the SM-1 culture in AWF resulted in a significantly higher maximum cell density than the isolate SR (**Figure 4C**), which correlates with the results of the competition assay in RT (Pool 09 in **Figure 3**), where SM-1 significantly outperformed SR.

**Figure 5:**
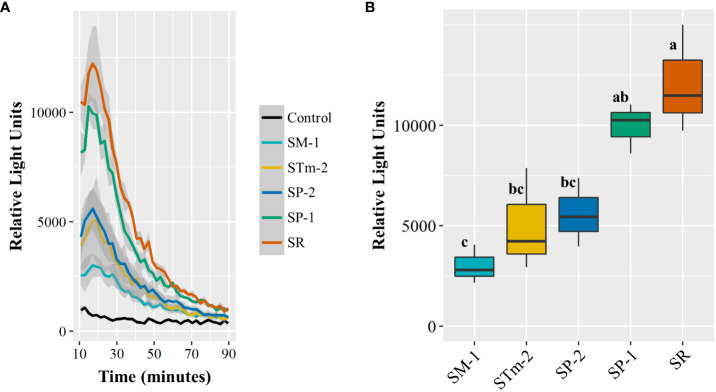
Reactive oxygen species (ROS) burst response of the lettuce cultivar Lollo Rossa to *Salmonella enterica* isolates collected from animal (SP-1, SP-2, and STm-1) and plant (SM-1 and SR) sources. ROS production over time as estimated by relative light units. Shaded areas represent the standard error **(A)**. The ROS burst curve peak (approximately at 18 minutes after elicitation) was used to assess statistical differences among the *S. enterica* strains **(B)**. Each ROS burst assay was conducted with 16 leaf discs per treatment and the experiment was performed three times with independent batches of plants (n=3). Plot center lines show the medians; box limits indicate the 25^th^ and 75^th^ percentiles, and whiskers extend to minimum and maximum data points. Different letters on top of adjacent boxes indicate significant statistical differences among the mean peak heights as calculated with ANOVA followed by Tukey’s test (α=0.05).

Next, we assessed the capacity of those isolates to induce plant immune responses via ROS burst. As we have previously shown that the *S. enterica* isolate STm 14028s induces a strong ROS burst in LR, but not in RT (Jacob and Melotto, 2020) and the total bacterial population of Pool 09 showed a neutral net growth in LR (**Supplementary Figure S2A**), we used this lettuce cultivar for this assay. The isolates induced significantly different ROS production in lettuce leaves; remarkably, SM-1 and SR evoked the smallest and the highest immune response, respectively (**Figure 5**). Altogether, these results suggest that these *S. enterica* isolates differ in their capacity to utilize nutrients available in the apoplast and to elicit the plant immune response. This variation might contribute to the differential ability of *S. enterica* strains to survive in a co-inhabited leaf apoplast niche.

## 4 Discussion

Some bacterial species have an outstanding environmental plasticity (Walters and Martiny, 2020), while others are highly host-adapted, such as *S. enterica* ser. Typhi (Hoffman and Luby, 2024). Moreover, certain species are able to cross the kingdom border, *e.g*., phytopathogens may impact human and animal health, while human pathogens might successfully reside in plants (Kim et al., 2020; Sobiczewski and Iakimova, 2022). Studies have increasingly shed light into the potential of human pathogenic bacteria to adjust to plant environments, by gaining and/or exploiting traits that enhance their ability to live in these niches (Holden et al., 2009; Warriner and Namvar, 2010; van Overbeek et al., 2014; Zarkani and Schikora, 2021). Our results demonstrate that various strains of *S. enterica* can differ in their ability to survive and compete in a co-inhabited lettuce foliar niche. While the genetic basis of these differences is not yet established, variation in the induction of ROS burst and potential utilization of nutrients in the apoplast may be plausible mechanisms.

Taking advantage of the lettuce genotypic variability, we used two contrasting lettuce cultivars, Red Tide (RT) and Lollo Rossa (LR) and observed that the *S. enterica* isolates used in this study overall tend to survive better on RT than LR, individually (**Figure 1**) or combined in pools (**Supplementary Figure S2**). This finding is in agreement with the previously described increased fitness of *E. coli* O157:H7 and *S. enterica* serovar Typhimurium 14028s on RT (Jacob and Melotto, 2020; Oblessuc and Melotto, 2020). It is possible that these bacterial pathogens of humans interact with the plant as commensals, occupying the phyllosphere while not benefiting or harming the host. Nevertheless, non-plant pathogenic microbes living in the leaf apoplast must avoid eliciting strong defense responses to survive in this niche (Reinhold-Hurek and Hurek, 2011). Therefore, lettuce cultivars lacking a broad basal immune response could provide a more suitable environment for *S. enterica* persistence. Variation in the colonization of the leaf by enteric human pathogens among lettuce genotypes might also be significantly impacted by differences in the phyllosphere microbiota (Lima et al., 2013; Leonard et al., 2021). Future research would be necessary to elucidate their potential roles in the phenotype observed in our study. It is important to note that a neutral or negative population growth, however, does not exclusively indicate lack of bacterial replication. In these scenarios bacterial cells could potentially replicate, but the plant immune responses and/or competition with other members of the microbiota could result in mortality that is faster than the replication in a population. In addition, bacterial population neutral net growth might reflect the survival of persister cells, however, the assays cannot distinguish between persisters and growth/death ratio. Nonetheless, bacterial enumeration is a good indicator of population dynamics and net growth over time. We also observed that the population net growth of the 14 *S. enterica* isolates varied significantly on both LR and RT (**Figure 1A**), revealing intraspecies differences within *S. enterica* in its ability to survive on lettuce. Thus, genotypic variation of *S*. *enterica* significantly affects the success of phyllosphere colonization, which has also been reported in alfalfa, fenugreek, lettuce, red cabbage, spinach, and tomato (Han and Micallef, 2014; Cui et al., 2018; Erickson and Liao, 2019; Wong et al., 2019). In particular, Wong et al. (2019) observed that the population growth of 43 *S. enterica* strains from 29 serovars varied from 1 to 7 log CFU/g on lettuce and tomato seedlings after 5 days of incubation. Although isolates from multiple animal and plant sources were included, no clear correlation between the type of previous niche and the ability to colonize the phyllosphere was reported (Wong et al., 2019). Similarly, we observed no evident association between the type of strain source, animal or plant, and the level of bacterial net growth after individual inoculations (**Figure 1A**). In addition, the relative bacterial net growth among the *S*. *enterica* isolates varied according to the lettuce genotype (**Figure 1B**).

A successful life of microbes in the phyllosphere depends on additional factors such as the environment (*i*.*e*., temperature, radiation) and interactions with other co-inhabitants (Vorholt, 2012). Studies have shown that interactions between *S. enterica* and members of the leaf microbial community can significantly impact, positively or negatively, the colonization outcome of this human pathogen (Brandl et al., 2013). Although the co-existence of different *Salmonella* serovars in the apoplast of lettuce leaves might be a rare event, competition assays *in planta* were intended to identify strains more adapted to the plant niche in a complex environment. Our assays demonstrated differential performance of *S. enterica* isolates cohabiting the intercellular leaf space and revealed isolates that have greater or lower relative capacity to compete in a shared lettuce phyllosphere (**Figures 2**, **3**; **Supplementary Figure S4**). It is unlikely that the expansion step required for barcode sequencing created bias in their relative abundance used to estimate competitive capabilities of the isolates as we observed no difference in the growth of isolates in LSLB medium (**Figure 4A**). The results from the competition assays indicate the change in the relative abundance of the isolates in the bacterial community over time, assessed by barcode sequence counts, and do not reflect the absolute values of their individual population size. Therefore, competition assay results are not directly comparable to bacterial population net growth (positive, neutral, or negative) of individual isolates calculated by CFU counts on plates. Nonetheless, these competition assays are still useful to estimate the relative fitness of each isolate in co-inoculated leaves. Competition assay outcomes might be the result of differential rates of cell persistence, proliferation, or death among the competing isolates. We observed that isolates from high-risk foods (SM-1 from lettuce and SO and SK from alfalfa sprouts) can perform equally well or outcompete isolates from the other sources, especially those collected from other plant-associated niches (**Figures 2**, **3**). In addition, *S*. *enterica* isolates collected from animal sources, except for SP-1, exhibited an overall good performance in the competition assays (**Figures 2**, **3**). Interestingly, in the paired competition assays (**Figure 2**) we were able to detect that SM-1 (serovar Montevideo, collected from head lettuce) significantly outcompetes SM-2 (serovar Montevideo, collected from tomato), whereas there is no statistical difference with SM-3 (serovar Montevideo, collected from cattle). Overall, these observations suggest that isolates collected from plant sources generally do not perform the best on lettuce as compared to other strains recovered from animals. Recent studies have shown that *S. enterica* fitness in different niches can be relevantly affected by pre-exposure to plant tissues. For instance, the growth of *S*. *enterica* ser. Typhimurium strain LT2 in lettuce-based medium enhances its capacity to persist in soil (Fornefeld et al., 2017). Furthermore, *S. enterica* cells collected from internalized populations in lettuce and green amaranth foliar tissues showed an improved acid tolerance, manifested by the increased surviving ability after 75 min at pH 2.7 (Grivokostopoulos et al., 2022). Acid tolerance as a physiological stress response of *Salmonella* to acidic food environments has been observed in fruit such as oranges (Eblen et al., 2004).

Nutrient acquisition in the leaf environment is crucial for the effective colonization of this niche by bacteria (Fatima and Senthil-Kumar, 2015). A recent study showed that differences in growth performance of *S*. *enterica* strains LT2 and 14028s in AWF collected from *Nicotiana benthamiana* are associated with mutations in the *rpoS* stress-response sigma factor gene in *S*. *enterica* LT2 (Lovelace et al., 2022). In particular, the *rpoS* modifications altered the utilization of L-malic acid, an abundant carbon source in *N*. *benthamiana* AWF (Lovelace et al., 2022). Our results show that the *S. enterica* isolates tested in Pool 09 (**Figure 3**) can grow differently in the AWF collected from RT (**Figure 4**). Interestingly, SM-1 showed a significantly higher cell density after incubation in AWF as compared to that of SR (**Figure 4**), observations that correlate with their competition phenotype in this lettuce cultivar (**Figure 3**). These findings suggest that the competition fitness might be, in part, due to a variation in the ability to utilize apoplastic nutrients and the potential to convert different types of metabolites (*i*.*e*., sugars) more readily to support the bacterial population net growth and/or to cope with the stress of a suboptimal environmental condition.

Several reactions of the plant defense repertoire have been previously reported to be induced by *S*. *enterica* Typhimurium 14028s and *E*. *coli* O157:H7 in lettuce, including ROS burst, callose deposition, and stomatal closure (Roy and Melotto, 2019; Jacob and Melotto, 2020). Transcriptomic profiling has also revealed the modulation of genes involved in ethylene, salicylic acid, and jasmonic acid signaling pathways and genes encoding pathogenicity-related proteins in the lettuce cultivar Tizian after inoculations with *S*. *enterica* Typhimurium 14028s (Jechalke et al., 2019). We observed that the induction of the ROS burst response in the lettuce cultivar LR varied among the assessed *S. enterica* isolates (**Figure 5**). Remarkably, the strains SR and SM-1 exhibited significant contrasting levels in the modulation of the lettuce defense response, showing correlation with their performance in the competition assay using the complex Pool 09 (**Figure 3**). Differential ROS burst responses and expression of plant defense marker genes in *Arabidopsis thaliana* have been previously shown to be associated with variations in the amino acid sequence of the flagellin epitope 22 of *S*. *enterica* Typhimurium 14028s and Senftenberg S05219 03 and of the phytopathogen *Pseudomonas syringae* pv. *tomato* DC3000 (Garcia et al., 2014). Overall, our results suggest that intraspecies variation in *S. enterica* isolates might impact the bacterial ability to cope with immune responses and to avoid recognition by the plant cells.

In summary, our research provides evidence for a significant variation among *S*. *enterica* isolates in their capacity to thrive in the leaf environment and highlights the relevance of studying isolates from fresh produce linked to disease outbreaks to identify strains exhibiting traits conferring enhanced leaf colonization. Human pathogenic bacteria with higher fitness on edible leaves, while preserving virulence on their natural human host, pose a serious threat to food safety, which should be mitigated. Future studies are key to elucidating the genetic components responsible for the variation among human pathogenic bacteria in their fitness in the foliar niche.

## Data availability statement

The original contributions presented in the study are included in the article/**Supplementary Material**. Further inquiries can be directed to the corresponding author.

## Author contributions

CJ: Data curation, Formal analysis, Investigation, Methodology, Writing - original draft, Writing - review & editing. JS: Formal analysis, Investigation, Methodology, Writing - review & editing. DFB: Writing - review & editing. WC: Investigation, Writing - review & editing. SP: Data curation, Formal analysis, Investigation, Methodology, Writing - review & editing. MMc: Conceptualization, Formal analysis, Funding acquisition, Project administration, Resources, Supervision, Writing - original draft Writing – review & editing. MM: Conceptualization, Formal analysis, Funding acquisition, Methodology, Project administration, Resources, Supervision, Writing - original draft, Writing – review & editing.
