# Supplementary figures and images for: Intraspecies competition among Salmonella enterica isolates in the lettuce leaf apoplast
Source: Front Plant Sci. 2024 Jan 30;15:1302047. doi: 10.3389/fpls.2024.1302047 (PMC10861783; doi:10.3389/fpls.2024.1302047)

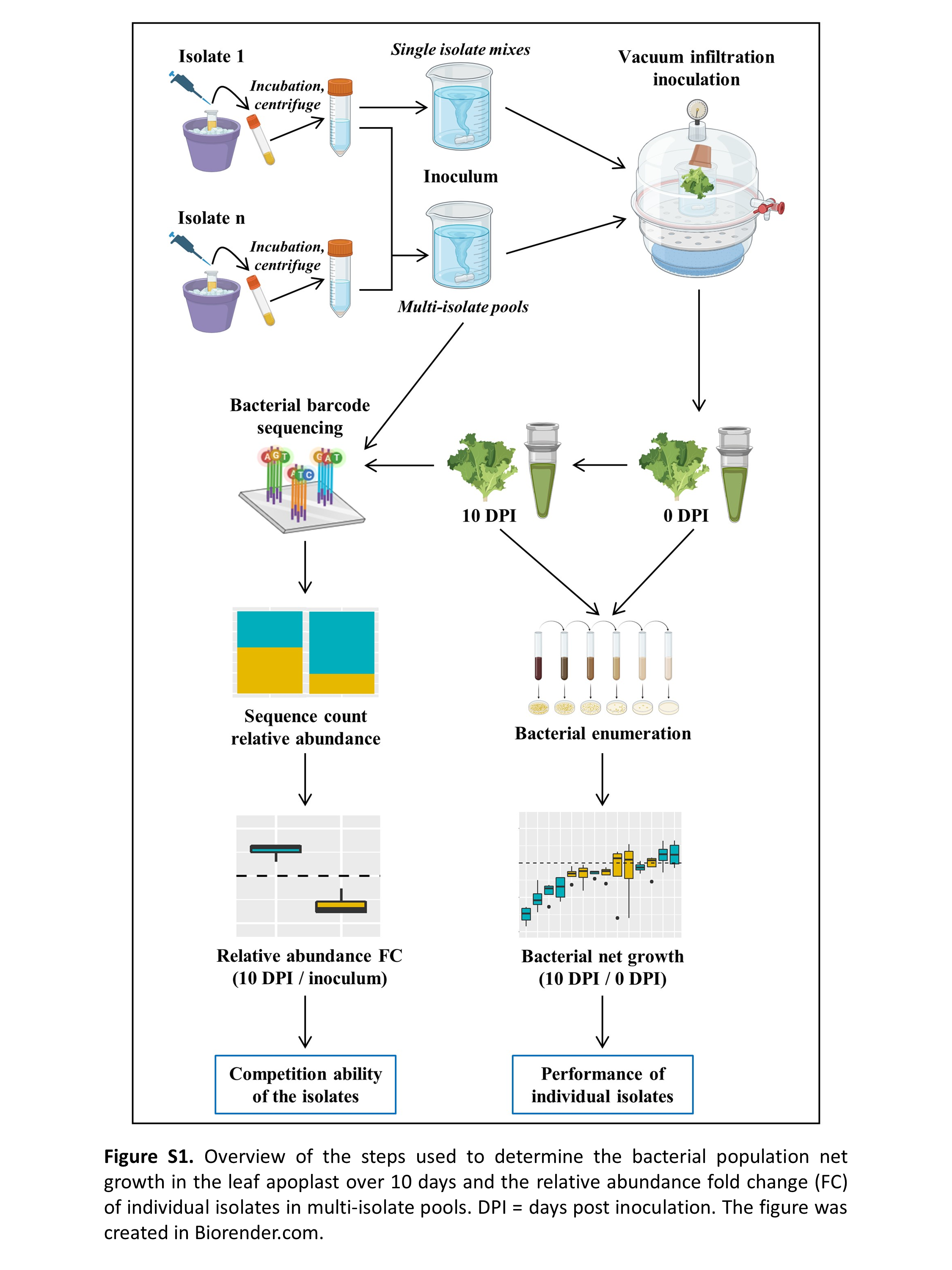

Supplement: Supplementary file 1 [file Image_1.tif]

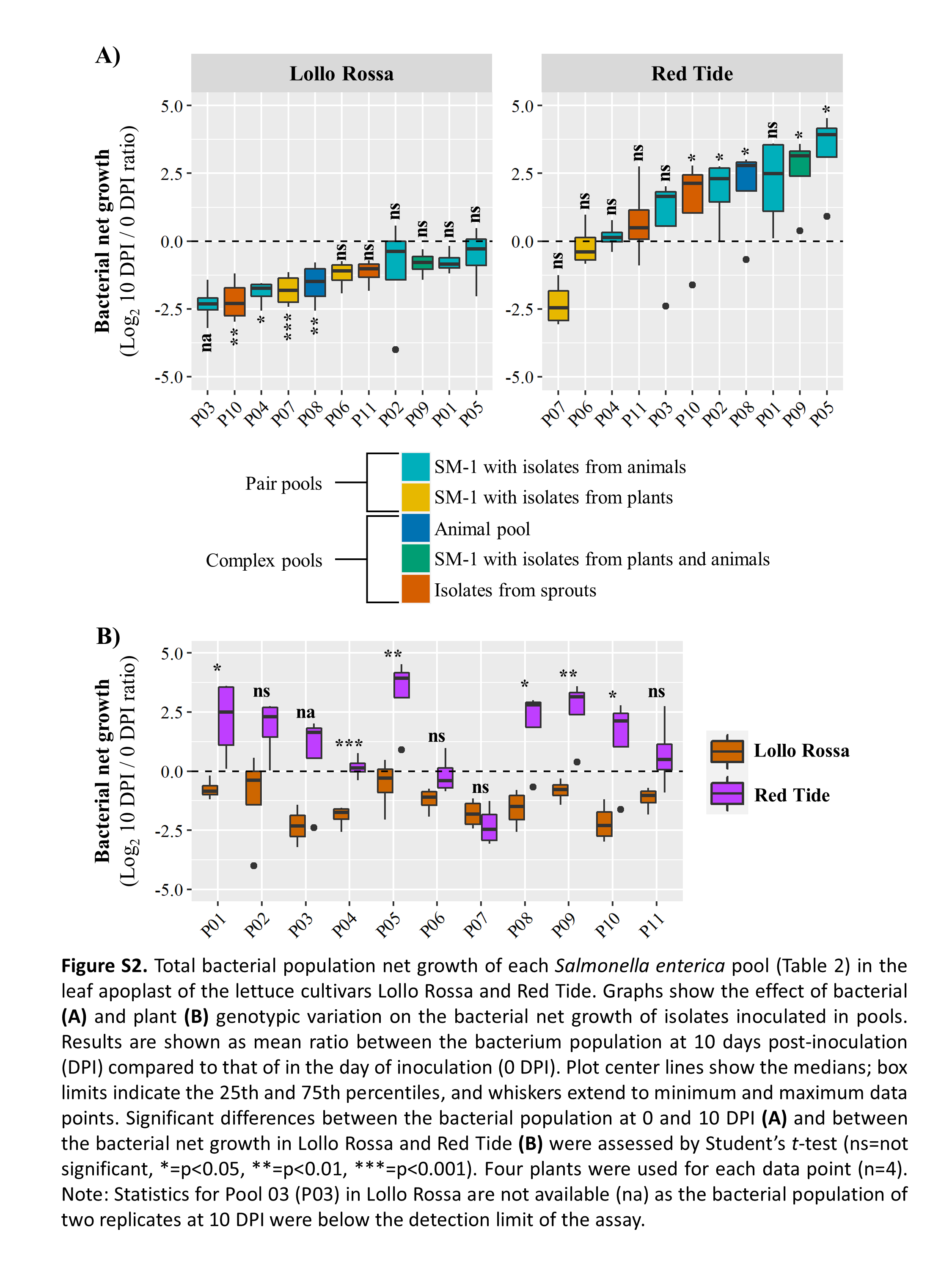

Supplement: Supplementary file 2 [file Image_2.tif]

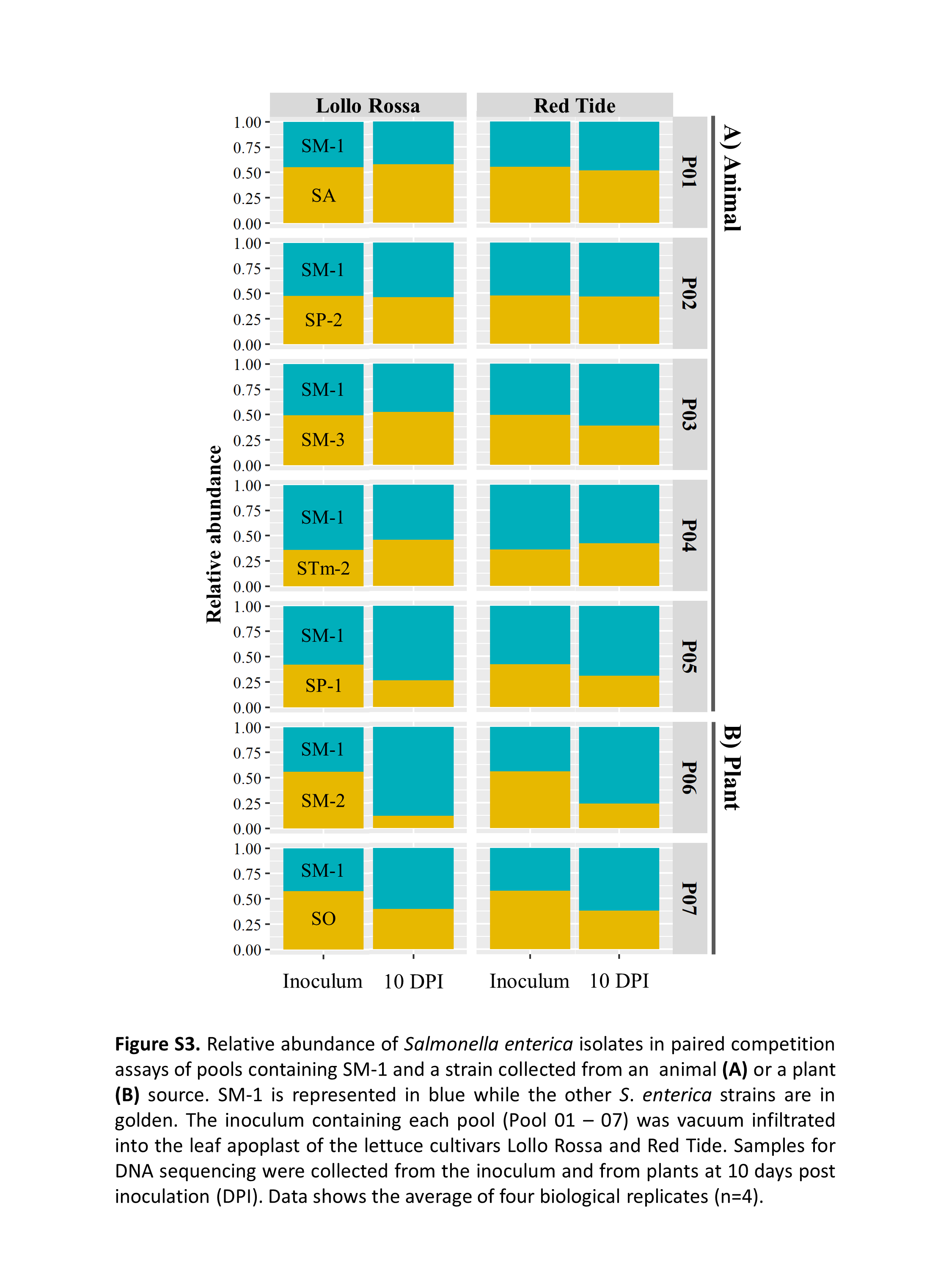

Supplement: Supplementary file 3 [file Image_3.tif]

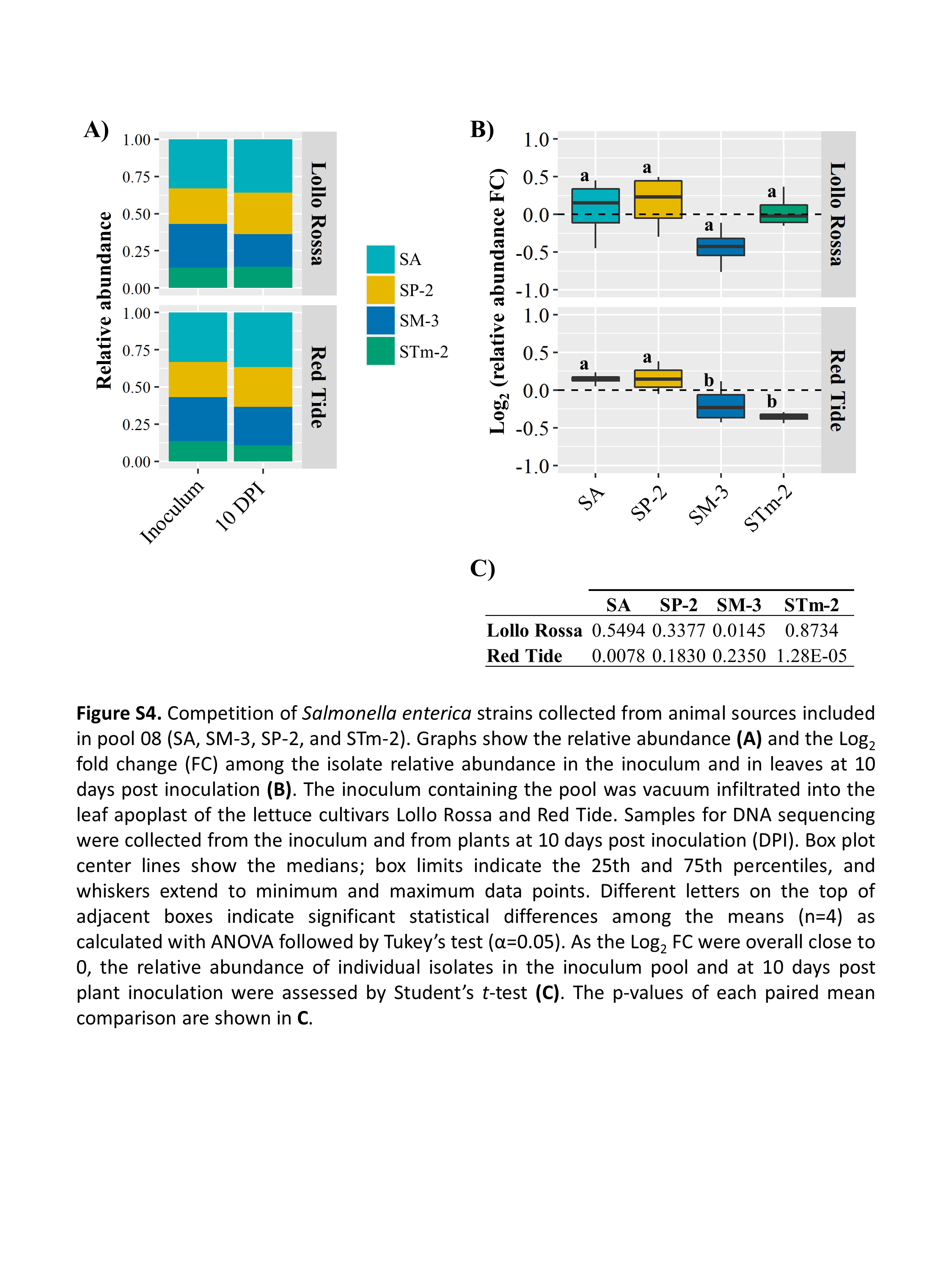

Supplement: Supplementary file 4 [file Image_4.tif]
